# Three Novel Lanthanide Metal-Organic Frameworks (Ln-MOFs) Constructed by Unsymmetrical Aromatic Dicarboxylatic Tectonics: Synthesis, Crystal Structures and Luminescent Properties

**DOI:** 10.3390/molecules190914352

**Published:** 2014-09-11

**Authors:** Ya-Pan Wu, Dong-Sheng Li, Wei Xia, Sha-Sha Guo, Wen-Wen Dong

**Affiliations:** College of Materils & Chemical Engineering, Collaborative Innovation Center for Microgrid of New Energy of Hubei Province, China Three Gorges University, Yichang 443002, China

**Keywords:** Ln (III) coordination polymers, crystal structure, topology, luminescent properties

## Abstract

Three novel Ln(III)-based coordination polymers, {[Ln_2_ (2,4-bpda)_3_ (H_2_O)*_x_*]·*y*H_2_O}_n_ (Ln = La (III) (**1**), *x* = 2, *y* = 0, Ce (III) (**2**), Pr (III) (**3**), *x* = 4, *y* = 1) (2,4-H_2_bpda = benzophenone-2,4-dicarboxylic acid) have been prepared *via* a solvothermal method and characterized by elemental analysis, IR, and single-crystal X-ray diffraction techniques. Complex **1** exhibits a 3D complicated framework with a new 2-nodal (3,7)-connected (4^2^·5) (4^4^·5^1^·6^6^·8) topology. Complexes **2** and **3** are isomorphous, and feature a 3D 4-connected (6^5^·8)-CdSO_4_ network. Moreover, solid-state properties such as thermal stabilities and luminescent properties of **1** and **2** were also investigated. Complex **1** crystallized in a monoclinic space group *P2_1_/c* with *a* = 14.800 (3), *b* = 14.500 (3), *c* = 18.800 (4) Å, β = 91.00 (3), *V* = 4033.9 (14) Å^3^ and *Z* = 4. Complex **2** crystallized in a monoclinic space group *Cc* with *a* = 13.5432 (4), *b* = 12.9981 (4), *c* = 25.7567 (11) Å, β = 104.028 (4), *V* = 1374.16 (7) Å^3^ and *Z* = 4.

## 1. Introduction

Lanthanide metal-organic frameworks (Ln-MOFs) have attracted ever-increasing interest not only because of their intriguing structural diversity, but also due to special photophysical properties [1,2,3,4]. However, due to their high coordination numbers and mutable coordination geometries, the assembly of lanthanide coordination polymers with specific geometry and properties might be uncontrollable [5], so designing and controlling lanthanide metal-organic frameworks (Ln-MOFs) with presupposed topological networks and functions remains a difficult and challenging task. According to the latest CCDC research (version 5.35), a number of aromatic multicarboxylate ligands such as benzene-dicarboxylate, benzenetricarboxylate, benzenetetracarboxylate, *etc.* have been extensively used to assemble fascinating structures with luminescent and atypical magnetic properties [6,7,8].

Among the various aromatic carboxylate ligands, rigid phenyl-, biphenyl- or polyphenyl- carboxylates with π-conjugated systems have been more extensively employed to provide a great variety of topological architectures with desired properties due to their remarkable versatile coordination modes [9,10]. By contrast, organic carboxylates with specific geometric configurations are poorly studied. More recently, we have focused on unsymmetrical semirigid aromatic dicarboxylatic ligand benzophenone-2,4-dicarboxylic acid (2,4-H_2_bpda) to construct new coordination polymers with different structures and properties. The ligand benzophenone-2,4-dicarboxylic acid (2,4-H_2_bpda) could freely bend and rotate to meet the requirements of coordination geometries of metal ions in the assembly process [11]. To our knowledge, so far no more than twenty examples of transition metal coordination polymers based on the 2,4-H_2_bpda ligand have been reported [11,12,13,14,15,16], and no Ln (III)-bpda coordination polymers have been synthesized and researched. However, the lanthanide cations exhibit different characteristic photoluminescent emissions in the ultraviolet or visible region. Herein, we report the synthesis, crystal structure of three new Ln (III)-based coordination polymers, {[Ln_2_ (2,4-bpda)_3_ (H_2_O)*_x_*]·*y*H_2_O}_n_ (Ln=La (III) (**1**), *x* = 2, *y* = 0, Ce (III) (**2**), Pr (III) (**3**), *x* = 4, *y* = 1,) (2,4-H_2_bpda = benzophenone-2,4-dicarboxylic acid). Moreover, the thermogravimetric analysis and luminescent properties of **1** and **2** were also discussed.

## 2. Results and Discussion

### 2.1. Crystal Structure Descriptions

#### 2.1.1. Structure of [La_2_ (2,4-bpda)_3_ (H_2_O)_2_]_n_ (**1**)

**Figure 1:**
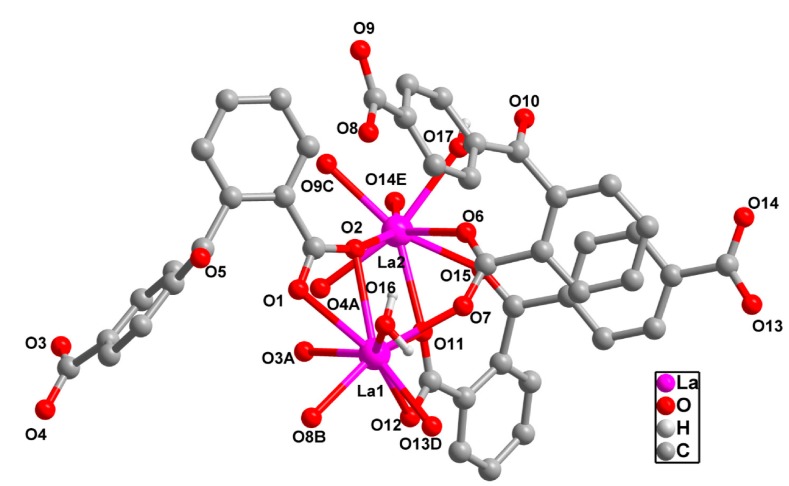
The coordination environment of La (III) center of **1**. Symmetry codes, A: −*x*, −*y* + 2, −*z*; B: −*x*, −*y* + 1, −*z*; C: −*x*, *y* + 1/2, −*z* + 1/2; D: −*x* + 1, −*y* + 1, −*z*; E: −*x* + 1, *y* + 1/2, −*z* + 1/2. All H atoms are omitted for clarity.

X-ray diffraction analysis reveals that **1** is a new 3D 2-nodal (3,7)-connected topological network based on infinite [La_2_(COO)_4_(H_2_O)_2_] chain SBUs. The asymmetric unit of **1** contains two crystallographically independent La (III) ion, three fully deprotonated 2,4-bpda^2−^ ligands and two coordinated water molecules. As shown in Figure 1, the La1 (III) is nine-coordinated by eight O atoms from six 2,4-bpda^2−^ carboxylate groups and one O atom from a coordinated water molecule, forming a distorted monocapped squareantiprism geometry. The La2 (III) is of a distorted bicapped triangle prism geometry surrounding by seven O atoms of six 2,4-bpda ligands and one O atoms of one cordinated water molecule. The La-O bond distances range from 2.401 (3) to 2.689 (2) Å and the O-La-O bond angle varies from 48.81 (7) to 153.75 (8).These bond lengths and angles all are comparable with those reported in the La^3+^-carboxylate compounds [17].

**Figure 2:**
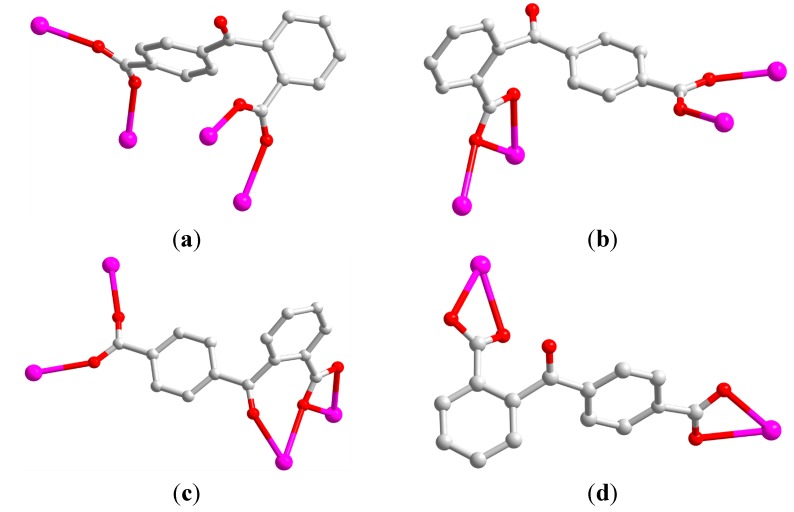
The coordination modes of 2,4-H_2_bpda ligand in complexes **1**–**3**. (**a**) μ_4_-η^1^:η^1^:η^1^:η^1^ mode; (**b**) μ_5_-η^1^:η^1^:η^2^:η^1^ mode; (**c**) μ_6_-η^1^:η^1^:η^2^:η^1^:η^1^ mode; (**d**) μ_2_-η^2^:η^2^ mode.

**Figure 3:**
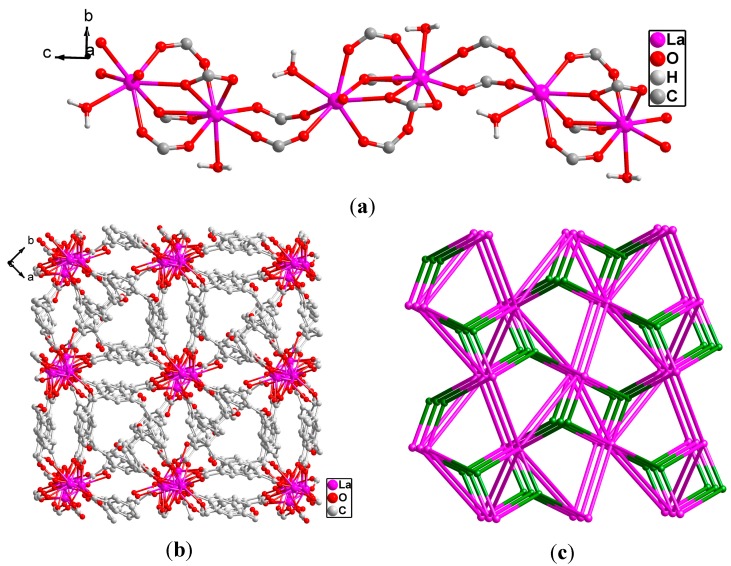
(**a**) View of the 1D [La_2_ (COO)_4_ (H_2_O)_2_] chain SBUs along the *c* axis; (**b**) View of the complicated 3D framework of **1** along the *b* axis. All H atoms are omitted for clarity; (**c**) Schematic description of a new 2-nodal (3,7)-connected topology with a point symbol of (4^2^·5) (4^4^·5^1^·6^6^·8), constructed from the 3-connected 2,4-H_2_bpda^2−^ and 7-connected binuclear La_2_ nodes (green: 2,4-H_2_bpda^2−^ pink: La_2_ nodes).

It is noted that three crystallographically independent 2,4-bpda ligands display three different coordination modes: μ_4_-η^1^:η^1^:η^1^:η^1^ (Type A, Figure 2a) and μ_5_-η^1^:η^1^:η^2^:η^1^ (Type B, Figure 2b) and μ_6_-η^1^:η^1^:η^2^:η^1^:η^1^ (Type C, Figure 2c), respectively. In **1**, the interlinkage between La (III) ions and carboxylate groups of bpda^2−^ generates an infinite La-carboxylate chain along the *c* axis (Figure 3a). Finally, these chains are extended by different oriented 2,4-bpda ligands to generate a complicated 3D frameworks (Figure 3b). Topologically, if binuclear [La_2_ (COO)_4_ (H_2_O)_2_] unit is considered as a 7-connected node, the type B and type C 2,4-bpda ligands are viewed as a linker and the type A 2,4-bpda ligands are viewed as 3-connected nodes. Hence, the structure of **1** can be best regarded as a new 2-nodal (3,7)-connected topology with a point symbol of (4^2^·5) (4^4^·5^1^·6^6^·8) (Figure 3c).

#### 2.1.2. Structure of {[Ce_2_ (2,4-bpda)_3_ (H_2_O)_4_]·H_2_O}_n_ (**2**)

**Figure 4:**
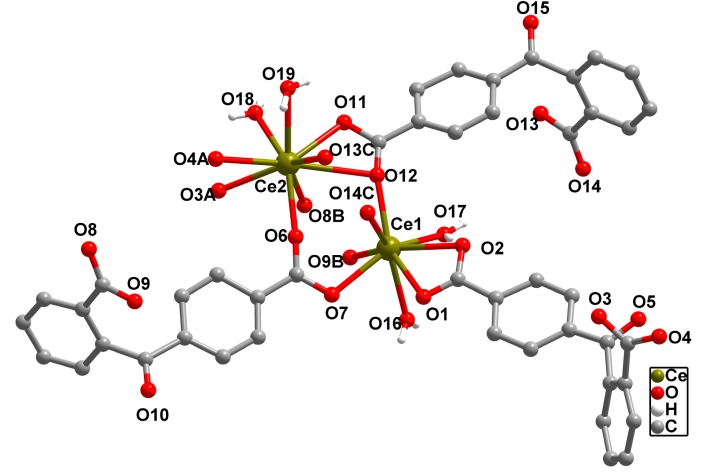
The coordination environment of Ce (III) center of **2**. Symmetry codes, A: *x* + 1/2, −*y* + 1/2, *z* + 1/2; B: *x* + 1/2, *y* + 1/2, *z*; C: *x* − 1/2, *y* − 1/2, *z*. All H atoms are omitted for clarity.

The complex **2** and **3** are isostructural and feature similar 3D framework; herein, only the structure of **2** will be discussed in detailed as a representation. X-ray crystallography reveals that complex **2** is of the monoclinic *C_c_* space group.The asymmetric unit of **2** contains two Ce (III) ions, three 2,4-bpda ligand, two coordinated water molecules and one free water molecule. Similar to complex **1**, the central Ce (III) ions have analogous coordination numbers. As shown in Figure 4, the eight-coordinated Ce1 (III) ion is distorted bicapped triangle prism geometry, which is completed by five carboxylic O atoms (O1, O2, O7, O9, O12, O14 ) from five 2,4-bpda^2−^ liands, two water oxygen atoms (O16 and O17) of coordinated water molecule. The La2 (III) shows is of a distorted bicapped triangle prism geometry [CeO_9_], which is ligated by seven oxygen atoms from five 2,4-bpda^2−^ liands and two water oxygen atoms of coordinated water molecule. The coordination Ce-O bonds, varying from 2.344 (3) to 2.608 (3) Å, are within the reported results [18]. 

**Figure 5:**
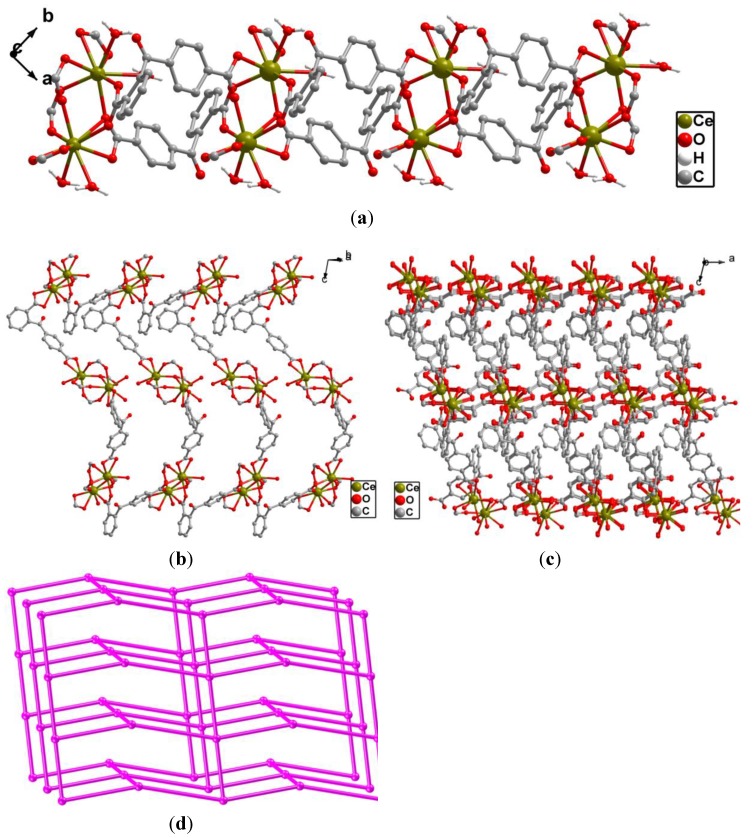
(**a**) View of the 1D [Ce_2_ (COO)_2_] binuclear chain SBUs in the *ab* plane; (**b**) The 2D wave-like layer structure in the *bc* plane; (**c**) View of the complicated 3D framework of **2** along the *b* axis. All H atoms are omitted for clarity; (**d**) Schematic description of the uninodal 4-connected (6^5^·8) CdSO_4 _topology, constructed from the 4-connected Ce_2_ node (pink: Ce_2_ nodes).

Although there are three different coordination modes in complex **2**, different from complex **1**, one new coordination mode μ_2_-η^2^:η^2^ (Type D, Figure 1a) was found. The two carboxylates of independent 2,4-bpda ligands adopt μ_2_-η^2^:η^2^ and μ_2_-η^2^:η^2^ coordination modes and link two Ce^3+^ centers to form a 1D [Ce_2_ (COO)_2_] binuclear chain SBUs with the Ce·Ce separation of 4.217 Å (Figure 5a). Each binuclear SBUs is coordinated by six 2,4-bpda ligands through the bidentate bridging, bidentate chelating, and monodentate coordination modes. Moreover, the 1D [Ce_2_ (COO)_2_] binuclear chain were extended in a 2D wave-like layer structure in the *bc* plane (Figure 5b). Finally, three kinds of 2,4-bpda ligands (type A, type B and type D) bridge adjacent four binuclear SBUs, which resulted in the complicated 3D networks (Figure 5c). From the view of topology, the [Ce_2_ (COO)_2_] binuclear SBUs can be simplified as 4-connected nodes and the 2,4-bpda ligands are viewed as a linker. And the whole framework forms a uninodal 4-connected CdSO_4_ net with the point symbol (6^5^·8) (Figure 5d).

### 2.2. Luminescent Properties

**Figure 6:**
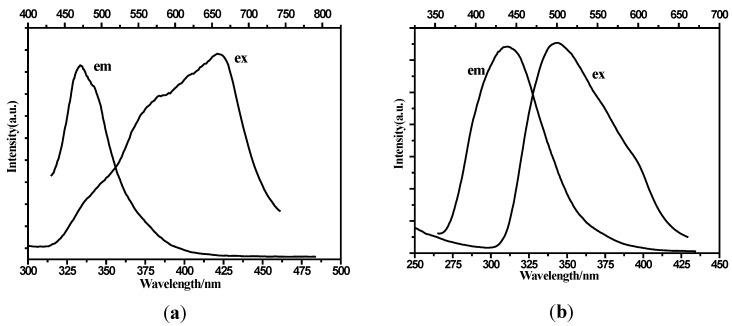
Fluorescence excitation and emission spectra of **1** (**a**) and **2** (**b**) in the solid state at room temperature. (λ_ex_ = 421 nm, λ_em_ = 471 nm for **1**; λ_ex_ = 344 nm, λ_em_ = 439 nm for **2**).

The solid-state fluorescent properties of complexes **1** and **2** at room temperature are shown in Figure 6. Complexes **1** and **2** display a fluorescent emission at around 471 nm (λ_ex_ = 421 nm) and 439 nm (λ_ex_ = 344 nm), respectively. As for the free organic ligands, a weak emission is observed at 394 nm (λ_ex_ = 280 nm) [14]. The crystal-field splitting of the 5d orbital was not observed in the two complexes because of the high coordination number of Ln (III) ions [17]. The emission spectra of complexes **1** and **2** are similar to that of the free 2,4-H_2_bpda ligand, indicating that the fluorescence of these two compexes is a ligand-based emission [19]. Compared with the free 2,4-H_2_bpda, the emission peaks of **1** and **2** have a visible red shift and their intensity is also increased, which could be due to intraligand π-π* or n-n* electron transitions [20]. The obvious enhanced intensities of complexes **1** and **2** could be attributed to the increased rigidity of the ligand after coordination to the Ln (III) center, which effectively reduced the loss of energy [21].

### 2.3. Thermogravimetric Analysis

**Figure 7:**
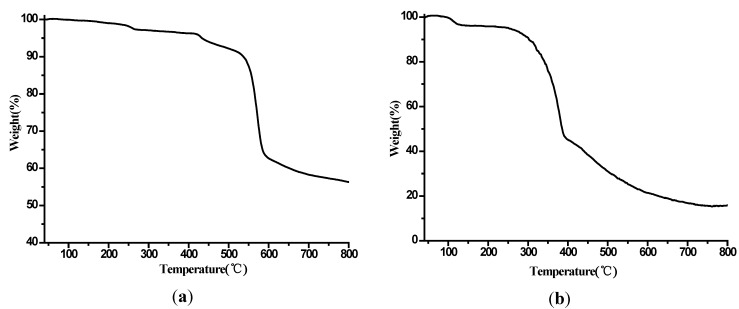
(**a**,**b**) Thermogravimetric curve of complexes **1** and **2**.

Thermogravimetric analyses (TGA) were monitored to observe the thermal behavior of complexes **1** and **2** (Figure 7). Complexes **1** and **2** showed similar thermal decomposition processes. Therefore, only the patterns of **1** will be discussed as an example. The first weight loss of 3.25% in the range of 50–150 °C is related to the loss of two coordinated water molecules (Calc. 3.22%). The residue is stable up to about 200 °C. After 300 °C, the network of **1** gradually collapses corresponding to the decomposition of organic components and the remaining residue is lanthanum oxide. 

## 3. Experimental 

### 3.1. Materials and Physical Measurements

The inorganic salts and organic regents were commercially available and used as supplied without further purification. The ligand benzophenone-2,4-dicarboxylic acid (2,4-H_2_bpda) was obtained from Alfa Aesar China Co. Ltd. (Beijing, China) and used as received. Elemental analysis for C and H were performed on a GmbH VarioEL V3.00 automatic elemental analyzer. The FT-IR spectra were recorded as KBr pellets with a Thermo Electron NEXUS FT-IR spectrometer in the 4000–400 cm^−1^ region. Thermogravimetric analysis was recorded with a NETZSCH STA 449C microanalyzer in air at a heating rate of 10 °C·min^−1^. Luminescence spectra for the solid samples were recorded with a Hitachi F-4500 fluorescence spectrophotometer at room temperature.

### 3.2. Synthesis of Complexes **1**–**3**

Single-crystal samples of complexes **1**–**3** suitable for X-ray analysis were obtained by a similar method to that described for complex **1**.

*[La_2_ (2,4-bpda)_3_ (H_2_O)_2_]_n_* (**1**). A mixture of La (NO_3_)_2_∙6H_2_O (0.0866 g, 0.2 mmol), 2,4-H_2_bpda (0.0268 g, 0.1 mmol), oxalic acid (0.0090 g, 0.1 mmol), NaOH (0.5 mL, 0.1 mol/L) and H_2_O (8 mL) was stirred under air atmosphere for 15 min and then sealed in a 25 mL Teﬂon-lined stainless steel vessel. After heating for 96 h at 160 °C, then the reaction system was cooled to room temperature and yellow block crystals of **1** were collected by filtration, washed with water and dried in air. Yield: 55%. Anal. Calc. for C_45_H_28_O_17_La_2_: C, 48.32; H, 2.52%; Found: C, 48.35; H, 2.50%. IR (KBr, cm^−1^) 3415 m, 3020 m, 1715 s, 1583 s, 1525 s, 1420 s, 1229 s, 1135 s, 847 m, 783 m, 725 m, 635 m.

*{[Ce_2_ (2,4-Bpda)_3_ (H_2_O)_4_]·H_2_O}_n_* (**2**). This complex was prepared in a similar method to that of **1** except for the fact La (NO_3_)_2_∙6H_2_O was replaced by an equivalent molar quantity of Ce (NO_3_)_2_∙6H_2_O. Yield: 52%. Anal. Calc. for C_45_H_34_O_20_Ce_2_: C, 46.00; H, 2.92%; Found: C, 45.85; H, 2.90%. IR (KBr, cm^−1^) 3425 m, 3012 m, 1735 s, 1573 s, 1521 s,1415 s, 1218 s, 1145 s, 830 m, 775 m, 710 m, 621 m.

*{[Pr_2_ (2,4-bpda)_3_ (H_2_O)_4_]·H_2_O}_n_* (**3**). This complex was prepared in a similar method to that of **1** except for the fact the La (NO_3_)_2_∙6H_2_O was replaced by an equivalent molar quantity of Pr (NO_3_)_2_∙6H_2_O. Yield: 48%. Anal. Calc. for C_45_H_32_O_20_Pr_2_: C, 46.02; H, 2.75%; Found: C, 46.05; H, 2.78%. IR (KBr, cm^−1^) 3410 m, 1725 s, 3018 m, 1580 s, 1519 w, 1425 s, 1225 s, 1120 s, 845 m, 785 m, 730 m, 627 m.

### 3.3. X-ray Crystallography

Single crystal X-ray diffraction analysis of **1**–**3** were collected on a Bruker SMART APEX II CCD diffractometer equipped with a graphite monochromated MoΚR radiation (λ = 0.71073 Å) by using φ/ω scan technique at 296 (2) K. The structures were solved by direct methods with SHELXS-97 [22]. A full-matrix least-squares reﬁnement on F^2^ was carried out using SHELXL-97 [22]. Absorption corrections were applied by using multi-scan program SADABS [23]. The hydrogen atoms were assigned with common isotropic displacement factors and included in the ﬁnal reﬁnement by use of geometrical restrains. Generally, the positions of C/N-bound H atoms were generated by a riding model on idealized geometries. The H atoms of coordinated water molecules of **1** and **2** were first located in difference Fourier maps, and then fixed in the calculated sites as riding. For **3**, the lattice water molecule was located at the special position and the affiliated H atoms were not determined. The crystallographic data and selected bond lengths and angles for **1 **and** 2** are listed in Table 1 and Table 2. 

**Table 1:** Crystal data and structure refinement parameters for complexes **1**–**3**.

	1	2	3
Empirical formula	C_45_H_28_O_17_La_2_	C_45_H_34_O_20_Ce_2_	C_45_ H_32_O_20_ Pr_2_
Formula weight	1118.49	1174.96	1174.53
Temperature	293 (2) K	293 (2) K	293 (2) K
Wavelength	0.71073	0.71073	0.71073
Crystal system space group	Monoclinic P2_1_/c	Monoclinic Cc	Monoclinic Cc
a (Å)	14.800 (3)	13.5432 (4)	13.533 (3)
b (Å)	14.500 (3)	12.9981 (4)	12.990 (3)
c (Å)	18.800 (4)	25.7567 (11)	25.768 (5)
α (°)	90	90	90
β (°)	91.00 (3)	104.028 (4)	104.22 (3)
γ (°)	90	90	90
Volume (Å^3^)	4033.9 (14)	4398.9 (3)	4391.3 (15)
Z Calculated density (Mg·m^−3^)	4, 1.842	4,1.774	4,1.777
Absorption coef. (mm^−1^)	2.170	2.127	2.276
F (000)	2192	2320	2320
Crystal size (mm)	0.20 × 0.17 × 0.14	0.32 × 0.27 × 0.23	0.50 × 0.35 × 0.30
θ range for data collection (°)	3.01–25.43	3.00–25.50	3.11–25.05
Limiting indices	−17 ≤ h ≤ 17 −17 ≤ k ≤ 17 −22 ≤ l ≤ 19	−16 ≤ h ≤ 16 −15 ≤ k ≤ 15 −21 ≤ l ≤ 31	−13 ≤ h ≤ 16 −14 ≤ k ≤ 15 −30 ≤ l ≤ 30
reﬂections collected/unique	37806/7406 [R (int) = 0.0341]	9876/6116 [R (int) = 0.0327]	19982/7245 [R (int) = 0.0306]
Max. and min.transmission	0.7509 and 0.6707	0.6405 and 0.5493	0.505 and 0.400
Refinement method	Full-matrix least-squares on F^2^	Full-matrix least-squares on F^2^	Full-matrix least-squares on F^2^
data/restraints/parameters	7406/0/577	6116/11/605	7245/11/617
Goodness-of-ﬁt on F^2^	1.079	1.196	1.010
R_1_^a^, wR_2_^b^ [I > 2sigma (I)]	0.0280, 0.0616	0.0552, 0.1341	0.0245, 0.0578
R indices (all data)	0.0316, 0.0635	0.0568, 0.1350	0.0255, 0.0589
Largest diff. peak and hole e.Å^−3^	0.963, −0.658	1.971, −2.656	0.837, −0.539

^a^*R*_1_ = Σ (|*F*_o_| − |*F*_c_|)/Σ|*F*_o_|; ^b^*wR*_2_ = [Σ*w* (*F*_o_^2^ − *F*_c_^2^)^2^/Σ*w* (*F*_o_^2^)^2^]^1/2^.

**Table 2:** Selected bond lengths (Å) and bond angles (°) for complexes **1**–**3**.

1		2		3	
La1-O8B	2.404 (2)	Ce1-O14C	2.417 (13)	Pr1-O1	2.454 (3)
La1-O13D	2.480 (2)	Ce1-O7	2.457 (12)	Pr1-O3A	2.441 (4)
La1-O7	2.487 (2)	Ce1-O16	2.504 (11)	Pr1-O6	2.417 (3)
La1-O3A	2.498 (2)	Ce1-O1	2.572 (12)	Pr1-O9B	2.408 (3)
La1-O1	2.582 (2)	Ce2-O8B	2.416 (10)	Pr1-O14C	2.479 (4)
La1-O12	2.577 (3)	Ce2-O11	2.506 (11)	Pr1-O13C	2.551 (4)
La1-O16	2.630 (3)	Ce2-O4A	2.526 (12)	Pr1-O16	2.505 (3)
La1-O2	2.689 (2)	Ce2-O19	2.561 (11)	Pr1-O17	2.557 (4)
La1-O11	2.688 (2)	Ce2-O12	2.866 (10)	Pr2-O2	2.429 (3)
La2-O9C	2.402 (3)	O4-Ce2	2.526 (12)	Pr2-O4A	2.414 (3)
La2-O4A	2.410 (2)	O9-Ce1C	2.501 (12)	Pr2-O6	2.822 (4)
La2-O14E	2.434 (2)	O14-Ce1B	2.417 (13)	Pr2-O7	2.478 (3)
La2-O6	2.454 (2)	Ce1-O12	2.427 (10)	Pr2-O8B	2.514 (4)
La2-O17	2.560 (3)	Ce1-O9B	2.501 (12)	Pr2-O11	2.529 (3)
La2-O2	2.560 (2)	Ce1-O2	2.516 (12)	Pr2-O12	2.529 (3)
La2-O15	2.586 (2)	Ce1-O17	2.595 (13)	Pr2-O18	2.548 (3)
La2-O11	2.605 (2)	Ce2-O6	2.455 (12)	Pr2-O19	2.566 (4)
O3-La1A	2.498 (2)	Ce2-O13C	2.506 (13)	O3-Pr1B	2.441 (4)
O4-La2A	2.410 (2)	Ce2-O3A	2.531 (12)	O4-Pr2B	2.414 (3)
O8-La1B	2.404 (2)	Ce2-O18	2.592 (12)	O8-Pr2A	2.514 (4)
O9-La2	2.401 (3)	O3-Ce2	2.531 (12)	O9-Pr1A	2.408 (3)
O13-La1D	2.480 (2)	O8-Ce2C	2.416 (10)	O13-Pr1	2.551 (4)
O14-La2	2.434 (2)	O13-Ce2B	2.506 (13)	O14-Pr1	2.479 (4)
O8B-La1-O13D	74.76 (8)	O14C-Ce1-O12	73.3 (4)	O9B-Pr1-O6	72.48 (12)
O8B-La1-O7	146.86 (8)	O12-Ce1-O7	122.2 (3)	O9B-Pr1-O3A	129.47 (11)
O13D-La1-O7	78.79 (8)	O12-Ce1-O9B	79.9 (4)	O6-Pr1-O3A	79.60 (12)
O8B-La1-O3A	81.85 (8)	O14C-Ce1-O16	141.7 (4)	O9B-Pr1-O1	79.78 (13)
O13D-La1-O3A	134.34 (9)	O7-Ce1-O16	74.4 (4)	O6-Pr1-O1	120.74 (11)
O7-La1-O3A	131.28 (8)	O14C-Ce1-O2	86.7 (4)	O3A-Pr1-O1	79.62 (12)
O8B-La1-O1	79.13 (8)	O7-Ce1-O2	127.0 (4)	O9B-Pr1-O14C	85.92 (14)
O13D-La1-O1	136.64 (9)	O16-Ce1-O2	86.8 (4)	O6-Pr1-O14C	101.33 (12)
O7-La1-O1	108.27 (8)	O12-Ce1-O1	143.8 (4)	O3A-Pr1-O14C	141.37 (13)
O3A-La1-O1	73.37 (8)	O9B-Ce1-O1	136.3 (4)	O1-Pr1-O14C	127.98 (12)
O8B-La1-O12	96.35 (8)	O2-Ce1-O1	51.6 (4)	O9B-Pr1-O16	142.41 (12)
O13D-La1-O12	70.19 (9)	O12-Ce1-O17	76.7 (4)	O6-Pr1-O16	144.93 (12)
O7-La1-O12	93.10 (9)	O9B-Ce1-O17	74.7 (4)	O3A-Pr1-O16	72.64 (12)
O3A-La1-O12	74.20 (9)	O2-Ce1-O17	67.7 (4)	O1-Pr1-O16	75.11 (13)
O1-La1-O12	147.57 (9)	O8B-Ce2-O6	72.7 (4)	O14C-Pr1-O16	87.92 (14)
O8B-La1-O16	81.94 (9)	O6-Ce2-O11	124.3 (4)	O9B-Pr1-O13C	80.67 (13)
O13D-La1-O16	75.75 (10)	O6-Ce2-O13C	76.3 (4)	O6-Pr1-O13C	143.48 (13)
O7-La1-O16	72.39 (9)	O8B-Ce2-O4A	124.4 (4)	O3A-Pr1-O13C	136.89 (12)
O3A-La1-O16	139.01 (9)	O11-Ce2-O4A	144.2 (4)	O1-Pr1-O13C	76.74 (12)
O1-La1-O16	66.71 (9)	O8B-Ce2-O3A	73.5 (4)	O14C-Pr1-O13C	51.56 (12)
O12-La1-O16	145.04 (10)	O11-Ce2-O3A	129.3 (4)	O16-Pr1-O13C	66.72 (13)
O8B-La1-O2	126.76 (8)	O4A-Ce2-O3A	51.5 (4)	O9B-Pr1-O17	134.60 (12)
O13D-La1-O2	151.47 (7)	O6-Ce2-O19	141.6 (4)	O6-Pr1-O17	77.37 (13)
O7-La1-O2	73.89 (7)	O13C-Ce2-O19	67.6 (4)	O3A-Pr1-O17	75.22 (12)
O3A-La1-O2	72.31 (8)	O3A-Ce2-O19	116.3 (4)	O1-Pr1-O17	145.56 (13)
O1-La1-O2	49.39 (7)	O6-Ce2-O18	146.4 (4)	O14C-Pr1-O17	67.54 (13)
O12-La1-O2	118.90 (7)	O13C-Ce2-O18	136.9 (4)	O16-Pr1-O17	75.18 (13)
O16-La1-O2	88.15 (9)	O3A-Ce2-O18	67.8 (4)	O13C-Pr1-O17	106.65 (13)
O8B-La1-O11	139.67 (8)	O8B-Ce2-O12	70.8 (4)	O4A-Pr2-O2	73.30 (12)
O13D-La1-O11	104.25 (9)	O11-Ce2-O12	48.3 (3)	O4A-Pr2-O7	76.62 (13)
O7-La1-O11	66.45 (8)	O4A-Ce2-O12	158.3 (3)	O2-Pr2-O7	124.40 (11)
O3A-La1-O11	70.41 (8)	O19-Ce2-O12	97.9 (3)	O4A-Pr2-O8B	131.44 (11)
O1-La1-O11	117.95 (8)	O14C-Ce1-O7	79.7 (4)	O2-Pr2-O8B	76.60 (12)
O12-La1-O11	48.81 (7)	O14C-Ce1-O9B	129.8 (4)	O7-Pr2-O8B	90.55 (13)
O16-La1-O11	137.79 (9)	O7-Ce1-O9B	80.0 (4)	O4A-Pr2-O12	124.23 (12)
O2-La1-O11	72.19 (7)	O12-Ce1-O16	144.9 (3)	O2-Pr2-O12	91.50 (12)
O9C-La2-O4A	80.18 (10)	O9B-Ce1-O16	72.7 (4)	O7-Pr2-O12	143.72 (12)
O9C-La2-O14E	76.44 (9)	O12-Ce1-O2	101.5 (4)	O8B-Pr2-O12	93.42 (11)
O4A-La2-O14E	87.56 (9)	O9B-Ce1-O2	140.8 (4)	O4A-Pr2-O11	73.29 (11)
O9C-La2-O6	121.04 (9)	O14C-Ce1-O1	80.5 (4)	O2-Pr2-O11	82.69 (12)
O4A-La2-O6	132.95 (8)	O7-Ce1-O1	75.6 (4)	O7-Pr2-O11	130.43 (13)
O14E-La2-O6	135.64 (8)	O16-Ce1-O1	66.1 (4)	O8B-Pr2-O11	138.60 (12)
O9C-La2-O17	82.72 (10)	O14C-Ce1-O17	135.3 (4)	O12-Pr2-O11	51.38 (11)
O4A-La2-O17	158.67 (9)	O7-Ce1-O17	145.0 (4)	O4A-Pr2-O18	142.50 (12)
O14E-La2-O17	76.06 (9)	O16-Ce1-O17	75.2 (4)	O2-Pr2-O18	141.79 (12)
O6-La2-O17	67.47 (8)	O1-Ce1-O17	107.2 (4)	O7-Pr2-O18	70.90 (12)
O9C-La2-O2	80.49 (8)	O8B-Ce2-O11	75.8 (4)	O8B-Pr2-O18	67.90 (11)
O4A-La2-O2	76.23 (9)	O8B-Ce2-O13C	130.4 (4)	O12-Pr2-O18	77.33 (12)
O14E-La2-O2	153.75 (8)	O11-Ce2-O13C	91.6 (5)	O11-Pr2-O18	115.16 (11)
O6-La2-O2	68.07 (7)	O6-Ce2-O4A	91.3 (4)	O4A-Pr2-O19	81.80 (13)
O17-La2-O2	113.38 (9)	O13C-Ce2-O4A	93.6 (4)	O2-Pr2-O19	145.77 (13)
O9C-La2-O15	145.33 (8)	O6-Ce2-O3A	82.8 (4)	O7-Pr2-O19	69.81 (12)
O4A-La2-O15	108.74 (10)	O13C-Ce2-O3A	138.8 (4)	O8B-Pr2-O19	137.33 (13)
O14E-La2-O15	70.72 (8)	O8B-Ce2-O19	142.6 (4)	O12-Pr2-O19	83.32 (12)
O6-La2-O15	78.08 (8)	O11-Ce2-O19	70.8 (4)	O11-Pr2-O19	67.66 (12)
O17-La2-O15	78.93 (10)	O4A-Ce2-O19	78.6 (4)	O18-Pr2-O19	69.90 (13)
O2-La2-O15	133.91 (7)	O8B-Ce2-O18	83.4 (5)	O4A-Pr2-O6	71.46 (11)
O9C-La2-O11	146.07 (8)	O11-Ce2-O18	69.4 (4)	O2-Pr2-O6	78.07 (11)
O4A-La2-O11	70.99 (9)	O4A-Ce2-O18	83.0 (4)	O7-Pr2-O6	48.17 (10)
O14E-La2-O11	118.66 (8)	O19-Ce2-O18	69.6 (4)	O8B-Pr2-O6	65.60 (11)
O6-La2-O11	71.23 (8)	O6-Ce2-O12	78.1 (3)	O12-Pr2-O6	158.11 (11)

Symmetry codes: **1**: A: −*x*, −*y* + 2, −*z*; B: −*x*, −*y* + 1, −*z*; C: −*x*, *y* + 1/2, −*z* + 1/2; D: −*x* + 1, −*y* + 1, −*z*; E: −*x* + 1, *y* + 1/2, −*z* + 1/2; **2**: A: *x* + 1/2, −*y* + 1/2, *z* + 1/2; B: *x* + 1/2, *y* + 1/2, *z*; C: *x* − 1/2, *y* − 1/2, *z*; **3**: A: *x* + 1/2, *y* − 1/2, z; B: *x* − 1/2, *y* + 1/2, *z*; C: *x* − 1/2, −*y* + 1/2, *z* − 1/2.

CCDC 1008351, 1008352 and 1008366 contain the supplementary crystallographic data of complexes **1**, **2** and **3** for this paper. These data could be obtained free of charge via www.ccdc.cam.ac.uk/conts/retrieving.html (or from the CCDC, 12 Union Road, Cambridge CB2 1EZ, UK; fax: +44 1223 336033; E-mail: deposit@ccdc.cam.ac.uk).

## 4. Conclusions 

In summary, three novel Ln (III)-based coordination polymers, {[Ln_2_ (2,4-bpda)_3_ (H_2_O)*_x_*]·*y*H_2_O}_n_ (Ln = La (III) (**1**), *x* = 2, *y* = 0, Ce (III) (**2**), Pr (III) (**3**), *x* = 4, *y* = 1,) have been prepared via a solvothermal method and characterized. The structural analysis indicates complexes **1**, **2** and **3** show a novel 3D 2-nodal (3,7)-connected (4^2^·5) (4^4^·5^1^·6^6^·8) topological network and a 3D 4-connected (6^5^·8)-CdSO_4_ net. Complexes **1** and **2** exhibit intense fluorescent emission in the solid state at room temperature upon photoexcitation. To the best of our knowledge, these three complexes represent the first Ln (III)-based coordination polymers constructed using the unsymmetrical semi-rigid 2,4-bpda ligand. The results will enrich current rare earth coordination chemistry and provide new insights into its application in engineering such Ln-MOFs with different structures and properties.
